# Supplementary material for: Ceftolozane-Tazobactam Treatment of Hypervirulent Multidrug Resistant Pseudomonas aeruginosa Infections in Neutropenic Patients
Source: Microorganisms. 2020 Dec 21;8(12):2055. doi: 10.3390/microorganisms8122055 (PMC7767535; doi:10.3390/microorganisms8122055)
Supplement: Supplementary file 1 [file microorganisms-08-02055-s001.pdf]

Supplementary

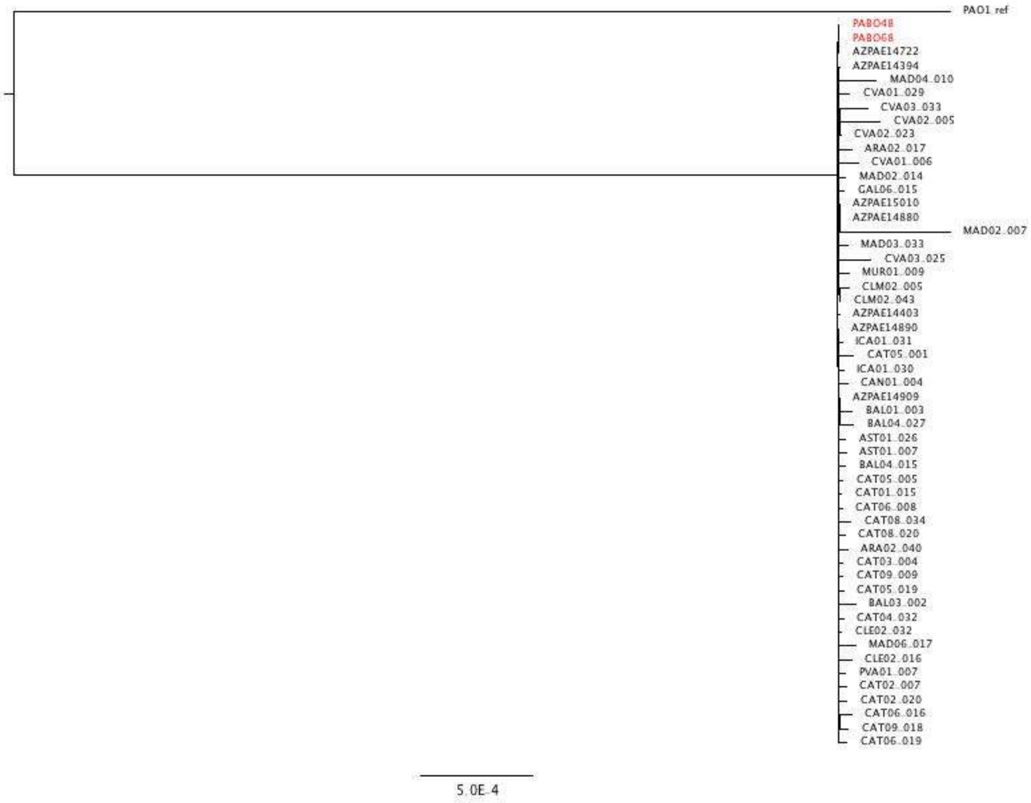

Figure S1. Maximum likelihood phylogenetic tree based on the SNPs in the core genomes of ST175 *Pseudomonas aeruginosa* clinical strains. Strains associated with transmission event described in this study are highlighted in red.

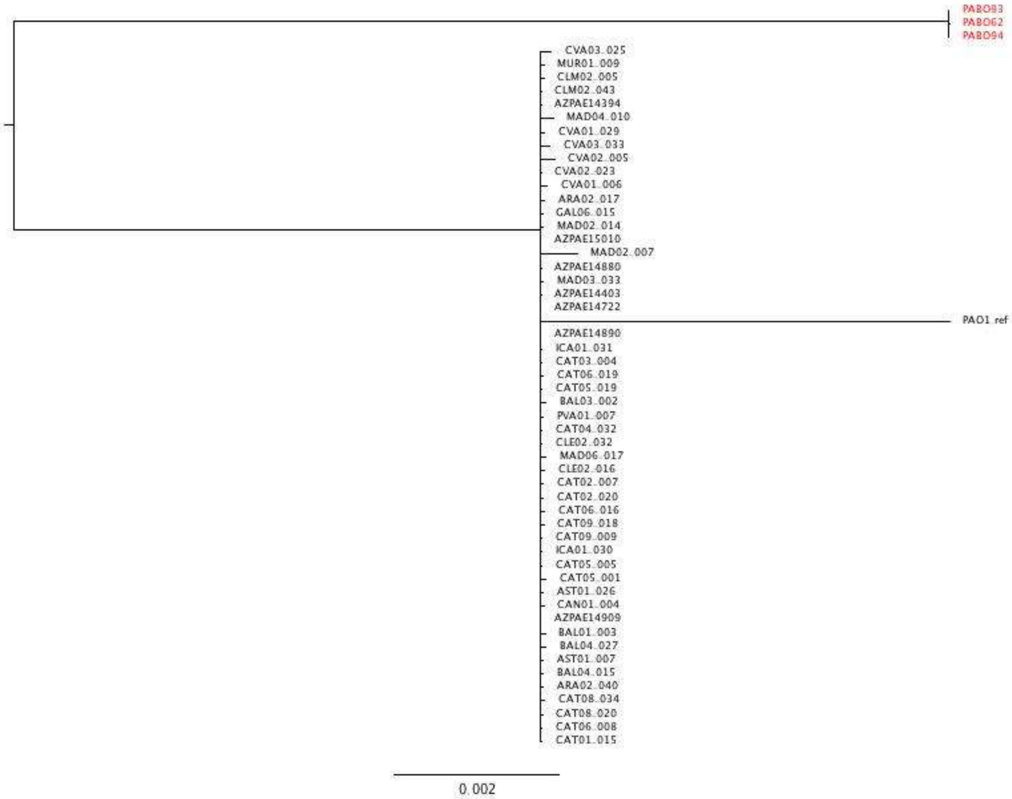

Figure S2. Maximum likelihood phylogenetic tree based on the SNPs in the core genomes of ST235 *Pseudomonas aeruginosa* environmental strains. Strains associated with transmission event described in this study are highlighted in red.
